# Review of the Undergraduate Psychiatry Curriculum: Integrating Themes of Culture, Diversity and Environmental Sustainability

**DOI:** 10.1192/bjo.2024.278

**Published:** 2024-08-01

**Authors:** Kiran Pindiprolu, Elizabeth Ajibade, Stefan McKenzie, Lauren Fitzmaurice, Reem Abed

**Affiliations:** Sheffield Health and Social Care, Sheffield, United Kingdom

## Abstract

**Aims:**

Integrated Learning Activities (ILAs) are a teaching method in Sheffield Medical School for Phase 3a students, where students will be in their third to fourth year of study and building clinical experience and skills. They're a flipped classroom technique, meaning students should arrive prepared to provide most input and clinicians are session facilitators. Each ILA has associated learning outcomes and reading material, and one or more case scenarios are used in sessions from which questions are generated for students to discuss. There are nine ILAs covering core psychiatric presentations, for example psychosis, and self-harm. Following student feedback, it became apparent that key themes around sustainability, diversity and culture were either not evident enough or used improperly. The project aimed to review the ILAs and associated documents to update and diversify the curriculum and integrate the above themes.

**Methods:**

Feedback was initially obtained from medical students and representatives from student societies on ILAs, with one scenario receiving strong feedback on its use of cultural themes. Further meetings with students were held, and the one scenario was collaboratively rewritten and rolled out across South Yorkshire.

A feedback survey was sent out regionally to gather facilitator feedback on the format and content of existing ILAs, and thoughts on adding content on sustainability and transcultural issues. Expressions of interest were called for from facilitators across the region, medical students and student societies, and patient experts to create a working group to review all ILAs and associated documents, with a view to diversify the curriculum and incorporate themes on culture, diversity and sustainability.

Two working groups successfully took place with diverse representation from each invited group. All ILAs and scenarios were reviewed, and these themes were able to be added using different techniques such as ensuring scenarios include cases from diverse backgrounds, removing descriptions of race and gender when not relevant, adding learning objectives on transcultural mental health issues and the impact of mental health on culture and vice versa, and adding scenarios and learning objectives on sustainability and sustainable practice.

Further surveys were generated for planned dissemination to students and facilitators for feedback which are planned for initial distribution in January 2024 onwards and results are awaited.

**Results:**

ILAs and associated documents were successfully reviewed allowing the curriculum to be diversified and updated. Due to the time constraints for project completion, it wasn't possible to have specialist input on gender and gender identity and so these themes were not able to be incorporated into the curriculum. Plans have been made for a further review to be conducted in approximately 12 months and these themes to be added at that time.

**Conclusion:**

This review has allowed for positive changes in the undergraduate curriculum and important issues around diversity, culture and sustainability and their impact on mental health and care are now specifically addressed. This aims to be the first of such collaborative curriculum reviews to ensure that the Psychiatry curriculum is up-to-date and fit to address emerging needs in mental health.

